# Multidrug Resistance of Cancer Cells and the Vital Role of P-Glycoprotein

**DOI:** 10.3390/life12060897

**Published:** 2022-06-15

**Authors:** Chenmala Karthika, Raman Sureshkumar, Mehrukh Zehravi, Rokeya Akter, Faraat Ali, Sarker Ramproshad, Banani Mondal, Priti Tagde, Zubair Ahmed, Farhat S. Khan, Md. Habibur Rahman, Simona Cavalu

**Affiliations:** 1 Department of Pharmaceutics, JSS College of Pharmacy, JSS Academy of Higher Education & Research, Ooty 643001, Tamil Nadu, India; ckhphd@jssuni.edu.in; 2 Department of Clinical Pharmacy Girls Section, Prince Sattam Bin Abdul Aziz University Alkharj, Alkharj 11942, Saudi Arabia; mahrukh.zehravi@hotmail.com; 3 Department of Global Medical Science, Wonju College of Medicine, Yonsei University, Wonju 26426, Gangwon-do, Korea; rokeyahabib94@gmail.com; 4 Department of Licensing and Enforcement, Laboratory Services, Botswana Medicines Regulatory Authority (BoMRA), Gaborone 999106, Botswana; frhtl6@gmail.com; 5 Department of Pharmacy, Ranada Prasad Shaha University, Narayanganj 1400, Bangladesh; ramproshad131135@gmail.com (S.R.); banani091110@gmail.com (B.M.); 6 Amity Institute of Pharmacy, Amity University, Noida 201303, Uttar Pradesh, India; tagde_priti@rediffmail.com; 7 Unit of Bee Research and Honey Production, Faculty of Science, King Khalid University, Abha 61413, Saudi Arabia; dzubair@gmail.com; 8 Research Center for Advanced Materials Science (RCAMS), King Khalid University, Abha 61413, Saudi Arabia; 9 Mahala Campus, Community College, King Khalid University, Abha 61413, Saudi Arabia; 10 Biology Department, Faculty of Sciences and Arts, King Khalid University, Dhahran Al Janoub, Abha 61413, Saudi Arabia; shakir.cgc@gmail.com; 11 Faculty of Medicine and Pharmacy, University of Oradea, P-ta 1 Decembrie 10, 410087 Oradea, Romania

**Keywords:** multidrug resistance, P-gp, cancer, inhibitors, clinical trials

## Abstract

P-glycoprotein (P-gp) is a major factor in the multidrug resistance phenotype in cancer cells. P-gp is a protein that regulates the ATP-dependent efflux of a wide range of anticancer medicines and confers resistance. Due to its wide specificity, several attempts have been made to block the action of P-gp to restore the efficacy of anticancer drugs. The major goal has been to create molecules that either compete with anticancer medicines for transport or function as a direct P-gp inhibitor. Despite significant in vitro success, there are presently no drugs available in the clinic that can “block” P-gp–mediated resistance. Toxicity, unfavourable pharmacological interactions, and a variety of pharmacokinetic difficulties might all be the reason for the failure. On the other hand, P-gp has a significant effect in the body. It protects the vital organs from the entry of foreign bodies and other toxic chemicals. Hence, the inhibitors of P-gp should not hinder its action in the normal cells. To develop an effective inhibitor of P-gp, thorough background knowledge is needed in this field. The main aim of this review article was to set forth the merits and demerits of the action of P-gp on cancer cells as well as on normal cells. The influence of P-gp on cancer drug delivery and the contribution of P-gp to activating drug resistance were also mentioned.

## 1. Introduction

The phenomenon known as drug resistance is responsible for the decrease in the restorative rate of most diseases as well as the loss in the therapeutic efficacy of most of the anticancer medications [1]. Treatment resistance is found not only to a single chemotherapeutic drug but to a diverse set of structurally and functionally distinct medicines. Long-term exposure to a single therapy and recurrent usage of the medicine leads to drug resistance, which reduces the therapeutic efficacy of the medications. Even if the dose is changed, there will be no significant alterations in this phenomenon. Drug tolerance occurs when a person develops resistance to medications [2]. This was often evident in two types of drug tolerance: pharmacokinetic drug tolerance and pharmacodynamic drug tolerance [3].

Following the drug’s entry into our bodies, dissolved into distinct segments, it is absorbed into the circulation and dispersed to numerous different places before it is finally expelled from the body. All these factors influence adverse effects and medication potency, as well as the duration of action. The primary cause of pharmacokinetic tolerance is when the medication fails to sustain its minimal therapeutic concentration at the target location [4]. In this scenario, the impact is produced by the enzymatic action of cytochrome P450 (CYP450). This kind of tolerance is mostly dictated by oral dose forms, which result in first-pass metabolism [5].

The development of pharmacodynamic tolerance occurs when cellular feedback to a substrate is focused. The primary cause of pharmacodynamic tolerance happens when the therapeutic concentration of the substrate to the binding receptors exceeds the maximal therapeutic concentration, resulting in receptor desensitization [6]. Other possibilities include a decrease in receptor density that is mostly related to the receptor agonist and a change in the action of the potential efflux rate. In most situations, drug resistance develops through repeated exposure to the drug, but immediate tolerances have been found in rare circumstances [3].

Anticancer drug resistance, anti-human immunodeficiency virus (HIV) drug resistance, anti-tubercular drug resistance, antibiotic resistance, anti-malarial drug resistance, and anti-microbial drug resistance are all common. The majority of medication resistance is mediated by multidrug resistance (MDR) proteins, where P-glycoprotein (P-gp) is the potent member of the MDR family and plays a significant role in lowering therapeutic effectiveness in most treatments [7]. The role of P-gp is to protect the cells by preventing the entrance of xenobiotics and harmful chemicals. However, because it is over-expressed in cancer cells, inhibiting medication access, its action should be reduced in such circumstances for therapeutic effectiveness [8].

Cancer cells develop chemotherapeutic resistance as a result of MDR. P-gp (also called MDR1), MDR-associated protein 1 (MRP-1), and breast cancer resistance protein (BCRP) [9] are all associated with MDR [10]. There are 48 energy-dependent membrane transporter proteins in the ATP-binding cassette efflux pump family (ABC). All three of these proteins are members of the ABC family, which is the largest protein group [11]. It can be found on the surfaces of epithelial cells in the adrenal cortex, the placenta, the gastrointestinal tract (GIT), the hepatobiliary tract, and the renal tubules as well as the blood-brain barrier (BBB) membranes [12]. ABC transporters are required in physiologic conditions to remove lipids, sterols, micro peptides, and toxic substances from the cytoplasm [13].

P-gp is overexpressed in many tumour types, which consider chemotherapeutic drugs foreign bodies and efflux them out of the cancer cells, reducing the therapeutic concentration of drug insider the cancer cells [14]. Cytotoxic medicines that disrupt DNA replication processes, killing rapidly reproducing cancer cells, are particularly vulnerable to P-gp overactivity. Newer drugs, on the other hand, that inhibit cancer cell growth and spread by targeting biochemical processes also interact with Pgp. It seemed sensible to look for P-gp inhibitors that may help cancer patients overcome MDR. P-gp is considered a major MDR transporter with respect to its resistance to carcinoma chemotherapy [15]. In cellular testing, many substances were discovered to be effective as P-gp inhibitors. Verapamil, a calcium channel antagonist utilized as a racemic combination in the nanomolar concentration range for the treatment of cardiovascular disorders, is one of the most investigated first-generation inhibitors. Although second-, third-, and fourth-generation inhibitors that were less harmful and more effective were created, the notion was never tested in clinical settings (see Section 5) [16].

P-gp is considered a major MDR transporter with respect to its resistance to cancer chemotherapy [15]. Szackas and colleagues had formerly verified 118 compounds with identified putative mechanisms of action on NCI-60 cancer cell lines. According to the researchers’ findings, over 95% of the compounds had a negative association between drug sensitivity and P-gp expression [17]. Several projects are currently underway to develop new drugs that bypass or exert more continuous P-gp inhibition through the use of novel pro-drug compounds. Surprisingly, data collected in studies suggest that P-gp expression plays a major role in inflammatory immune cell subsets [18]. However, a complete understanding of P-gp and its contradictory role in cancer and immune cells remains elusive in the tumour microenvironment [19]. P-gp is a major factor in the multidrug resistance phenotype in cancer cells. It is a protein that regulates the ATP-dependent efflux of a wide range of anticancer medicines and confers resistance. Presently there are no drugs available that “block” P-gp-mediated drug resistance. The main aim of this review article was to put forth the merits and the demerits of the action of P-gp on cancer cells. The influence of P-gp on cancer drug delivery and the approaches to alter this mode of action are also mentioned. This manuscript reviews 117 articles and provides a complex survey of current literature dealing with this topic.

## 2. The Genetics of P-gp

The P-gp/ABCB1 gene is found on chromosome 7q21.12 and consists of 29 exons spread over a 209.6-kb genomic region. The messenger RNA (mRNA) is 4872 bp long (including the 5′ untranslated region) and encodes a 1280 amino acid protein with a molecular weight of 141 KDa [20]. To date, up to 1200 single nucleotide polymorphisms (SNPs) in the coding region have been reported (database viewed from NCBI-SNP in November 2019), each with a different effect on protein expression and activity. As a result, the three most studied SNPs in the P-gp protein-coding region are rs1045642 (3435T > C, Ser893Ala/Thr) as well as rs1128503 (1236T > C, Gly412Gly) [21]. More than two-thirds (72%) of the SNPs were discovered in intracellular and extracellular regions of P-gp, while only 28% were discovered in the TM domain. Because the amino acid sequence (Ile1145Ile) remains unchanged, the synonymous mutation (rs1045642, 3435T > C) has no effect on protein functionality [22]. The 3435C allele is expressed in a proportional amount in every population, with Africans expressing it more than Caucasians or Japanese. It is not commonly used as a synonym for silent mutation. According to Hoffmeyer et al., the 3435 TT genotype population had lower P-gp expression in digestive tract epithelial cells [23]. The 3435T allele, on the other hand, had higher mRNA transcript levels. There is a belief that this variation in gene expression is due to mRNA secondary structure instability, which involves significance in the transformed membrane insert, more periods for mRNA folding/unfolding throughout the translation, and tertiary structural alignment, thus causing variations in substrate affinity [24]. As a result, the 3435CC genotype has higher P-gp expression and function than its counterpart genotypes, which are either 3435CT or 3435TT. Patients with the 3435TT genotype are more likely to develop chemotherapy resistance than those with the 3435CC genotype, requiring less drug to kill cancer cells [25]. Immunosuppression was also more common in patients with the 3435TT genotype than in those with the 3435CC genotype, [26] as demonstrated by lower levels of interleukin-2 (IL-2) in their blood [27].

The 2677T allele, which codes for serine-893, is found in 2–65% of people of different ethnicities [18]. In African populations, the frequency of the homozygous 2677 GG genotype important to 893-Ala/Ala P-gp has been reported to be as high as 81%, compared with 10–32% in other demographics such as Asian, European, Native American, Mexican, and Indian populations. In addition to these SNPs, the 2677A allele with Thr-893 P-gp has been described as occurring at a low frequency of only 0–17% in several populations [28]. This nonsynonymous mutation-inducing SNP has received a great deal of attention, but the probable impact on P-gp expression and functionality is still unknown [29]. Similarly, in different populations, rs1128503 (1236T > C) with a synonymous mutation leading to Gly412Gly P-gp has a reported frequency ranging from 30 to 93% [30]. There have been no confirmed differences in pharmacokinetics and pharmacodynamics among the genotypes 1236CC/CT/TT and 1236CC/CT/TT [18].

## 3. P-gp Expression and Its Structure and Functions

**Figure 1:**
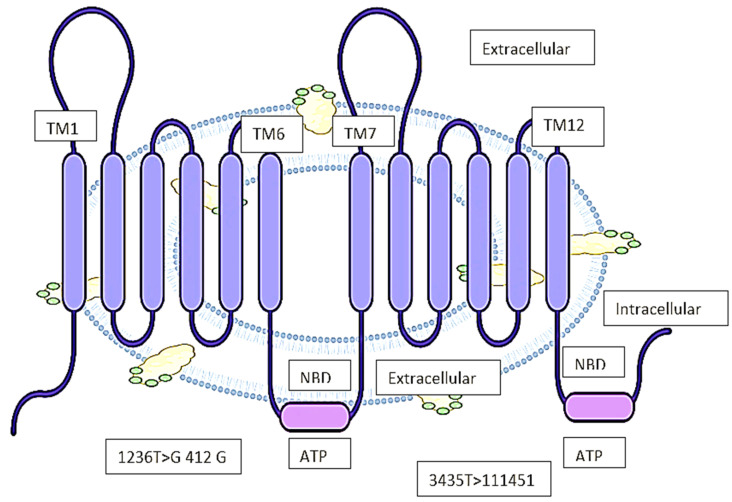
The structure of P-gp and its binding capacity. Abbreviations: TM–transmembrane, NBD–Nucleotide binding site, ATP–Adenosine triphosphatase.

Biedler et al. first proposed that MDR could be facilitated by a cell surface protein [31]. Schinkel et al. later demonstrated that ivermectin concentrations in brain tissue were 100-fold higher in the genetically engineered mice using a murine abcb1 (P-gp) knock-out model [32]. This gene’s mRNA transcript is 4872 bp long, with a 5′ untranslated region, and it encodes the 1280 amino acid P-gp protein. According to the Kyte-Doolittle hydropathy plot, the secondary structure has 12 transmembrane domains (TMD) (Figure 1) with numerous substitute transcripts and splice variants of unknown implications that have been conveyed through various literature. Post-translational modifications such as differential phosphorylation and N-glycosylation are believed to affect the functionality of P-gp [33]. The Pim-1 phosphorylation of S683 on glycosylated P-gp is also thought to cause multimerization and surface membrane stabilization. Although it has been demonstrated that two phosphorylation residues bind to tubulin, it has not been demonstrated that they are significant in persuading downstream signalling or protein activity. The 12 TMDs form a lipophilic [34] pore-like channel in the cell membrane to endorse the drug efflux of lipophilic and amphipathic complexes (Figure 1). Both ATP-binding domains are found on the cytoplasmic intracellular part of the protein. In 2009, the first high-resolution X-ray crystallography structure of mouse P-gp was reported, with 87% homology to human P-gp [35,36]. Further investigation at a resolution up to 3.3 revealed primarily comparable tertiary structural features. According to in silico structure–activity relationship (SAR) studies, P-gp can bind to stereoisomers of the same compound in different ways and has multiple binding sites to allow for the binding and efflux of multiple drug substrates. In early SAR studies, P-gp was difficult to use due to its high lipophilicity index, which rendered it water insoluble, and its high tertiary structural flexibility [37]. Alamet et al. recently revealed a 3.5-resolution structure using lipidic nanodiscs that allowed for a much better understanding of SAR biochemistry. P-gp has the canonical ABC transporter fold, with two pseudo-symmetric TMD, each half containing six TM helices, and one cytosolic domain with ATP-nucleotide binding functionality (NBD) [38]. NBD domains dimerize at the interface to bind and hydrolyse ATP, as do the NBD domains of many ABC proteins. A flexible linker of 60–70 amino acids with numerous phosphorylation sites attaches both pseudo-halves of P-gp. The cavity on the cytoplasmic side of the protein is 6000 square meters in size [39]. Drugs are supposed to enter this cavity over the portals in the cytoplasm and the internal leaflet of the membrane, then exit over the extracellular side, which has a pore size of 70–200 nm depending on protein orientation as explained in Figure 1. P-gp undergoes dynamic conformational changes to allow for a broad spectrum of substrate binding and efflux, which is connected to ATP binding and hydrolysis on the cytoplasmic side, allowing for unidirectional substrate superficial flow [40], while the inward V-conformation is the most energy-efficient. Thermodynamic studies show that at a high energy state with ATP-derived energy consumption, the passing outward in front of confirmation is adopted [18].

**Figure 2:**
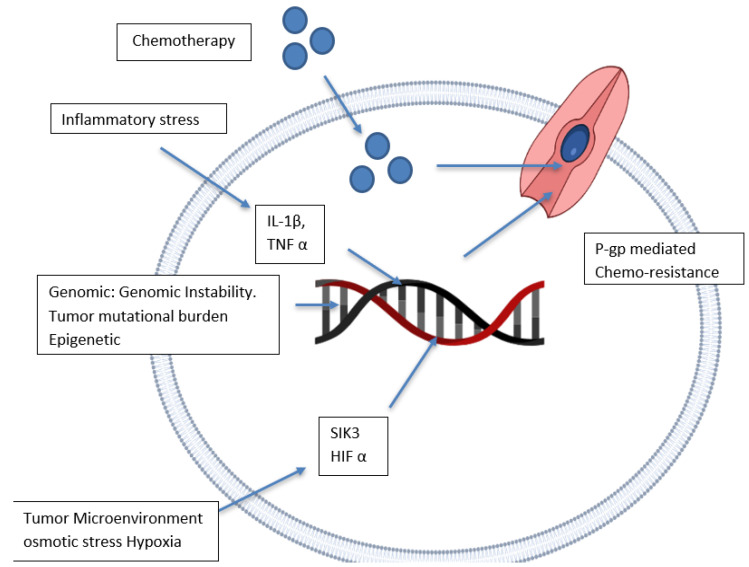
P-gp expression upregulation and its mechanism. Abbreviations: IL–interleukin, P-gp–P-glycoprotein, HIF–Hypoxia-inducible factor, SIK–Salt-inducible kinase, TNF–tumour necrosis factor.

There are disagreements about the location of the ATP-binding domain in the reported crystal structure due to the usage of detergents and the lack of nucleotides used to attain the crystal structure [41]. It is well documented that the two ATP-binding domains are on the intracellular side since cellular ATP concentration (1–10 mM) far surpasses the domain-binding persistence (0.01 mM) [42]. Irrespective of the debates over the P-gp tertiary membrane-bound structure, the crystal structure has been resolved, allowing for the identification of P-gp drug substrates and inhibitors in silico [43]. P-gp upregulation has been linked to a variety of factors, including epigenetic mechanisms, intrinsic cancer genomic instability, and inflammatory stressors in the tumour [44] microenvironment (Figure 2).

Several studies have shown that gene rearrangements and tumour mutational burden are significant mechanisms for controlling and modulating the abcb1 gene’s promoter region, which leads to its expression [45]. Ras, c-Raf, p53, and other oncogenes have all been linked to the regulation of P-gp expression [46]. The promoter region of the gene was found to be demethylated in various types of leukaemia with increased P-gp expression, implying that epigenetic modification plays a role in the activation of P-gp-mediated drug resistance [47]. According to studies, the activity of both acetyl-H3 and histone deacetylase increases after cancer chemotherapy. In the P-gp promoter region, acetyl-H3 was discovered to act as 968 bp upstream [18]. In MCF-7 breast cancer (BC) cell lines, transcription factors like CEBPb have been shown to induce P-gp expression. In MCF-7 and MDA-MB-231 BC cells, high salt-mediated osmotic stress (10.05 mM NaCl) increased intracytoplasmic calcium concentration by activating store-operated calcium entry (SOCE) from the endoplasmic reticulum [48]. Hypertonic stress increased P-gp expression, resulting in paclitaxel drug resistance in these BC cells. Hypoxic stress has been shown in some studies to increase P-gp expression by interacting with the P-gp promoter region via HIF1. In addition, P-gp-independent MDR has also been discovered in osteosarcoma cells [49,50,51].

## 4. The Kinetics of P-gp

The function of P-gp is the primary cause for multidrug resistance in cancer cells. P-gp moves a diverse spectrum of structurally and functionally distinct cytotoxic compounds out of the cell using the energy provided by ATP. Changing this efflux mechanism of P-gp is a critical technique for overcoming MDR and increasing therapeutic effectiveness throughout therapy. The incorporation of P-gp inhibitors with resistant medication may be beneficial in inhibiting P-gp activity [52]. The overexpression of P-gp in cancer cells relative to normal cells is the primary cause of MDR. P-gp decreases the minimal therapeutic concentration, which reduces the overall permeability of the medication for reaching the target site. P-gp is an essential component in the drug distribution process. It acts on the BBB as well as the placental barrier. It can have an influence on the distribution of certain therapeutic drugs while also reducing their activation in the body [53]. The involvement of P-gp in the brain has evolved to the point where it fights the entrance of neurotoxic medicines into the brain and hence maintains the penetration of central nervous system (CNS) agent delivery in the brain [54]. P-gp also protects the foetus against foreign bodies and hazardous chemicals entering the placenta [55].

The enzymatic activity of CYP3A4 and the efflux mechanism by P-gp both contribute to the lower therapeutic effectiveness and bioavailability of medicines taken orally. These are significant defence systems in the gut that serve as a protective barrier against the introduction of harmful chemicals and xenobiotics [56]. These proteins are mostly overexpressed in enterocytes and hepatocytes, and they also play a role in the first-pass metabolism of pharmaceuticals. As an example, piperine can be used to support this claim. Black pepper contains the flavonoid piperine. It has the capacity to function as a natural inhibitor of P-gp and CYP34A when co-administered and can boost the therapeutic efficacy of medications [1]. To induce this effect, the piperine dosage should be optimal and reach the minimal therapeutic concentration. Another example is the effect of grapefruit juice. When grapefruit juice and saquinavir are taken together, the therapeutic concentration of the parent medication can be enhanced when taken orally [57].

The mechanisms of renal excretion involve the processes of glomerular filtration, tubular secretion, and then reabsorption. P-gp is engaged in the efflux of xenobiotics and waste materials from our bodies through renal excretion, which results in a lower level of the therapeutic medicine in blood plasma. This can also be changed by combining flavonoids with medicinal drugs. When digoxin, a flavonoid, and cyclosporine A were administered together, cyclosporine increased digoxin levels in the plasma via lowering tubular secretion and the glomerular secretion rate [58]. When itraconazole and cimetidine are combined, comparable outcomes are produced. The impact of P-gp on medication excretion can also be modulated by utilizing natural P-gp inhibitors. When co-administered, quercetin, a natural P-gp inhibitor, can increase the therapeutic concentrations of a wide range of medicines in the target site [59]. In turn, therapeutic medicines such as azithromycin, erythromycin, cyclosporine A, and doxorubicin block biliary drug excretion mediated by P-gp [60].

P-gp plays a key role in decreasing medication bioavailability and distribution because it is overexpressed in the intestinal tract, which acts as a substrate for P-gp and limits its absorption route. As a result, the therapeutic level and bioavailability of the medications are not met. ATP binds to the cytoplasmic side of P-gp and triggers ATP hydrolysis, causing the substrate to be effluxes from the cell. The excretion of the substrate is followed by the release of the phosphate from the ATP molecule [61]. When adenosine diphosphate (ADP) is released, a new ATP molecule binds to the secondary ATP binding site. The protein will be reset after the hydrolysis and release of ADP and a phosphate molecule [62].

## 5. Substrates of P-gp and Its Drug Interaction

P-gp transports a wide range of substrates that differ structurally and functionally from one another. P-gp substrates generally appear to be lipophilic as well as amphipathic in nature. The majority of P-gp inhibitors are produced to modify its functioning [63]. The mechanism of action is either through competing with drug binding sites without interfering with ATP hydrolysis or through obstructing the ATP hydrolysis process. Recently, an allosteric mechanism for P-gp-mediated transport was added alongside the other two mechanisms [64]. Before being joined or transported to the extracellular membrane leaflets, P-gp substrates are attached to the protein molecule [65].

According to the research findings, P-gp has the capacity to interact with over 20 substrates or modulators. Anthracycline, fluorescent lipids, and vinca alkaloids are examples of substrates that are readily transported by P-gp. The binding effect of modulators such as cyclosporine and verapamil are used in chemotherapy to modulate P-gp activity. The P-gp binding pockets’ considerable flexibility and low specificity might be used to overcome MDR-related difficulties during chemotherapy [55]. P-gp inhibitors are classified primarily based on their specificity, affinity, and toxicity [64].

First-generation inhibitors: This group of inhibitors is pharmacologically active, and they are employed in particular treatments. Reserpine, cyclosporine A, verapamil, yohimbine, toremifene, quinidine, and tomoxifene are a few examples. In the case of leukemia cells, the resistance might be reversed by employing verapamil to produce an effective action. However, when a large dose of the medicine is administered to the patient, it causes cardiovascular toxicity. As a result of their lower therapeutic effectiveness, these inhibitors are being replaced with second-generation inhibitors [52].

Second-generation inhibitors: The substrates in this family of inhibitors are pharmacologically inactive in nature but act on P-gp. This generation of inhibitors is created by structurally altering first-generation inhibitors in order to achieve low toxicity, high selectivity, and potency. Doxverapamil, dofequidarfumerate, biricodar citrate (VX710) [66], valspodar (PSC 833) [67], and dexniguldipine are examples of this generation of inhibitors. This group mostly includes non-immunosuppressive equivalents of doxverapamil as well as cyclosporine A [68]. PSC 833, the most often used inhibitor, has a potency that is 5–10 times that of cyclosporine A. These inhibitors, on the other hand, have a higher affinity for and inhibitory action against ABC transporters and CYPA4 enzymes [69].

Third-generation inhibitors: Third-generation inhibitors are often created to alleviate the issues associated with first- and second-generation inhibitors. The key advantage of utilizing third-generation inhibitors is that they are less toxic than the previous two generations of inhibitors, and they are more selective and effective against P-gp [70]. They have no pharmacological interactions with chemotherapeutic drugs and have been discovered to be 200 times more powerful than the previous two generations of inhibitors. Zosuquidar (LY335979), mitotane (NSC-38721), laniquidar (R101933) [71], tariquidar (XR9576) [72], ONT-093, elacridar (F12091), annamycin, HM30181, R10933, and biricodar are other examples [73]. According to the 3D QSAR and QSAR activity, the structure of the inhibitors is mostly responsible for producing the inhibitory activities. According to studies, the inhibitory action is produced by the tariquidar’s heterocyclic ring near the antranilamide ring. However, new research indicates that tariquidar has both substrate and inhibitory effects on P-gp [74].

Natural inhibitors or fourth-generation inhibitors: Because of the toxicity and limited treatment options with synthetic inhibitors, natural inhibitors, including dietary supplements, are being investigated. Natural substances and food extracts have been shown to have an influence on P-gp to reverse MDR and to have anticancer properties [75]. Examples include curcumin, quercetin, piperine, capsaicin, and limonin [76].

The P-gp transporter has been shown to efflux a diverse range of molecules with varying chemical structures (cyclic, polar, nonpolar, linear, aromatic, linear-lipophilic) and molecular weights (250–4000 Da) (Table 1). Among the first P-gp competitive inhibitors discovered were cyclosporine-A and verapamil [77,78]. In a recent high-throughput screen of 10,804 compounds, Lee et al. discovered 90 substrates, 55 of which were novel. Despite widespread interest in developing small molecules used as P-gp inhibitors to combat MDR in cancer chemotherapy, the vast majority of previously discovered inhibitors have failed to pass Food and Drug Administration (FDA)-approved clinical-phase trials [79]. One of the foremost reasons for the drug’s failure to progress through clinical trials is tissue toxicity at the high dose produced by P-gp inhibition. Verapamil and cyclosporine-A, two of the first P-gp inhibitors tested in clinical trials, confirmed a low affinity for P-gp, and are shown to necessitate multiple micromolar plasma concentrations that result in unacceptable cardiac and also immunosuppressive side effects [80].

Tariquidar and zosuquidar, two newly optimized P-gp inhibitors, were created to increase potency (10–100 nM) and P-gp specificity. However, there appears to be some conflicting evidence in the literature regarding tariquidar’s role as an ATPase inhibitor or enhancer, as well as its role as a P-gp substrate or inhibitor [81]. Tariquidar, according to Weidner et al., is neither an inhibitor nor a substrate of the human or mouse P-gp model [82]. The tertiary structure of P-gp lacks a well-defined ligand-binding pocket, making it difficult to design extremely specific competitive inhibitors for P-gp, unlike enzyme-substrate interactions such as the lock–and–key model or the induced-fit model. Some bivalent inhibitors, such as reversible dimers of quetiapine and prodrug dimers of paliperidone, that can bind to multiple P-gp interaction sites, have been designed to provide better inhibition than monovalent binding with limited success. The dose required for clinical human applications will be reduced if a drug design strategy is used that improves the specificity and potency of previously recognized and natural compounds with identified P-gp interaction [83]. Epothilones have been proposed as low-specificity P-gp substrates because they inhibit cell division via microtubules and have a chemical structure parallel to taxanes. When combined with capecitabine, ixabepilone (azaepothilone B), an analog of epothilone B, is effective in treating anthracycline- as well as taxane-resistant metastatic BC [84]. Using a semisynthetic analog of taxanes, several new cancer drugs have been developed that outperform paclitaxel [85].

Cabazitaxel (an FDA-approved drug) and ortataxel (another FDA-approved drug) have both been demonstrated to be effective in the treatment of hormone-resistant metastatic prostate cancer [86]. These compounds have been shown to be less susceptible to P-gp efflux. Synthesizing analogues of vinca alkaloids, such as taxanes, has received a great deal of attention [87]. Vinflunine, a fluorinated semisynthetic analogue of vinblastine, had a 2- to 13-fold lower susceptibility to P-gp-mediated efflux compared with that of vincristine and vinblastine [88]. As a result, vinflunine was approved as a second-line treatment for urothelial cancers in Europe in 2009. Correspondingly, an isoindoline urea derivative of vinblastine (at the same chemical position C20) was revealed to have a 100-fold higher cytotoxic potential, in contrast with vinblastine-resistant cancer cell lines [89]. In a similar vein, in cancer cell lines, an aryl amide derivative of vinblastine is less susceptible to P-gp-mediated efflux. The therapeutic efficacy of these drugs, on the other hand, has yet to be proven in clinical settings [63]. The use of liposomes to deliver doxorubicin was approved by the FDA in 1995 [90]. Other ionic and block copolymer-based drug alterations are still being investigated. The FDA has already approved Abraxane, albumin-bound paclitaxel, for the treatment of metastatic BC [91]. A poly-L-glutamic acid-paclitaxel combination of Opaxio/Xyotax is recently being tested in ovarian and oesophageal cancer Phase III clinical trials. CPMs and ADCs (antibody-drug conjugates) have long been used to deliver drugs to specific cells with minimal side effects [92]. In resistant cancers, octa arginine-conjugated taxol has been widely studied, and the goal of these conjugation techniques was to improve drug internalization. On the other hand, the efficacy of these conjugation techniques in lowering P-gp-mediated efflux and drug concentration in the cytoplasm is debatable [93]. The FDA approved gemtuzumab ozogamicin for a limited time, but the approval was later revoked due to the lack of a better overall survival profile. Preclinical studies have shown that CD33-conjugated maytansine is effective, but the clinical benefit has yet to be established [94]. P-gp efflux appears to have been approved by the FDA for the treatment of refractory Hodgkin’s lymphoma and systemic anaplastic large cell lymphoma [95].

## 6. Tumour Immunity and P-gp Function 

Phenotype switch, immune cell activation, and cytokine release are all linked to P-gp expression. P-gp expression is low in peripheral circulating monocytes but high in anti-inflammatory M82 tissue macrophages that infiltrate tumours [96]. P-gp expression is linked to dendritic cell maturation and activation, as well as their ability to present antigens professionally. Inhibiting P-gp with valspodar slowed DC maturation, as evidenced by the lower expression of the activation markers CD80 and CD40 [97]. The highest level of P-gp surface expression of any innate immune cell has been linked to their downstream cytotoxic functionality, with increased Fas-mediated (Fas/FasL) surface binding of P-gp NK cells to target cells resulting in the release of inflammatory cytotoxic secretory granules and target cell apoptosis [98]. Depending on the cell type, P-gp expression has different functions in adaptive immune cells. P-gp expression is associated with lymph node cell migration as well as a transitional phenotype in B cells. P-gp expression is associated with the inflammatory Th1/Th17 effector phenotype in CD4+T cells, but it is very low in anti-inflammatory Tregs [99]. The memory phenotype (IL18Ra+CD161+CD62Llo) is associated with P-gp expression in CD8+T cells. These P-gp-expressing CD8+ memory T cells in mucosal-related T-cells (such as those found in the GIT) have bidirectional responses through an original defensive role to avoid xenobiotic toxins, but when the normal microbiome is disrupted, they can improve effector responses, foremost to autoimmune diseases like Crohn’s disease and ulcerative colitis [100].

Increased MAPkinase/ERK signalling is linked to increased P-gp expression in myeloid and lymphoid lineage cells of AML and B-cell lymphomas [101]. Anti-CD20 and anti-CD19 monoclonal antibodies (mAb) therapy may be able to overcome P-gp-mediated chemoresistance, possibly since mAb cannot be effluxed by P-gp [102]. Only a few cases of P-gp expression in infiltrating immune cells in solid organ tumours have been discovered so far [96]. The frequency of P-gp-expressing mucosal-derived CD8+T cells in tumour tissue specimens was found to be higher in human colorectal cancer studies. On the other hand, the precise function of CD8+T cells is unknown. In addition, the chemoresistance of cancer cells in the tumour microenvironment may influence immune cell infiltration [103]. The phenotype of CD4+CD161+P-gp+ T cells increased in AML patients receiving long-term chemotherapy. Furthermore, the expression of the T-cell exhaustion markers PD-1 and CTLA-4 was reduced in this subset of CD4+helper-T cells. It has been demonstrated that Th17 and Th1 CD4+T-helper cell subsets secrete anti-tumour inflammatory cytotoxic cytokines such as IL-17, IFNg, TNFa, and granzyme [72,104,105]. P-gp-expressing CD4+T cells (CD4+CD73+T cells) were found to secrete more anti-cancer cytokines in tumour-infiltrating breast and ovarian carcinomas. Due to the chemical inhibition of P-gp, these T-lymphocytes were unable to secrete these cytotoxic cytokines via vesicular secretion [106]. As a result, using P-gp inhibitors in this situation is not advised because they may reduce the cytotoxic potential of tumour-infiltrating anti-cancer Th1 and Th17 CD4+T cell phenotypes. P-gp expression is also found in pro-tumour M82 macrophages as well as anti-tumour NK-cell and Th17/CD4+T cell subsets, implying that P-gp plays a seemingly contradictory role in tumour immunology [107].

## 7. P-gp and Clinical Trial Reports

Various clinical trials are being conducted to determine the effect of chemotherapeutic drug resistance on cancer cells due to the effect of P-gp [108]. The current trials in this area are given in detail in Table 2.

## 8. Authors’ Perspective

P-gp is an active member of the ATP-binding cassette (ABC) protein subfamily, which effluxes a wide range of therapeutic drugs from cells, resulting in multidrug resistance [14]. However, it has an amazing protective effect on normal cells and the outflow of dangerous and foreign compounds [109]. As a result, P-gp efflux is a vital step to overcome for successful therapy and drug development. Modifying P-gp function using various inducers, inhibitors, or genetic polymorphisms is a common strategy [110].

P-gp expression is found in all areas of the body, functioning as a protective barrier against the entrance of poisons and xenobiotics. Its action is unavoidable in the blood–placental barrier, blood–brain barrier, and blood–testes barrier, but when the focus is on the therapeutic aspect of the drugs, the absorption of the drugs through the intestinal lumen is slowed due to the expression of the P-gp in the intestinal lumen, although their presence is predicted all over the body; their expression is higher, however, in diseased cells, particularly cancer cells [109]. The plasma membrane of the intestinal epithelial cells pumps back drugs that enter it, are recognized as substrates, and are expelled. Higher quantities are seen in the biliary epithelium and the kidney’s proximal tubules, and the medicines are found in the bile and urine. P-gp inhibitors are being developed to suppress its role [12]. Though the inhibitors demonstrate effect when tested in preclinical investigations, their action slows when tested in clinical trials.

One of the most common causes of multidrug resistance is the overexpression of P-gp or closely similar ABC-transporter family members. Unfortunately, none of the previously discovered and investigated P-gp inhibitors as potential co-therapeutics for MDR diseases have proven their efficacy in clinical studies [107]. Recent studies using the P-gp inhibitor tariquidar have indicated toxicity, and several phase III trials have been halted. In recent phase III research, another P-gp inhibitor, zosuquidar, was not demonstrated to improve outcomes in persons with advanced acute myeloid leukaemia [111]. Despite these obstacles, a recent randomised phase III research including high-risk acute myeloid leukaemia patients discovered that blocking P-gp can improve cancer patient outcomes. According to these findings, the beneficial effects of inhibitors were particularly noticeable in those with elevated P-gp expression [42]. P-gp appears to be an essential and vital target for therapeutic discovery and development, despite its poor clinical success. Recent advances in understanding the structure and mechanism of P-gp and the related drug pumps will surely aid in the rational development of pump-specific inhibitors [112]. Many previously investigated inhibitors are believed to bind to drug binding sites and to limit chemotherapeutic export by competing for transport cycles as P-gp transport substrates. Because of this property, extremely large systemic dosages were almost certainly necessary, which resulted in off-target damage [113].

P-gp is the major game changer in the field of drug discovery, associated not only with cancer but also with the majority of diseases, which include tuberculosis, malaria, and many antibiotic-related resistances. There are in vitro and in vivo methods developed for the determination of the P-gp response to various chemicals and agents. P-gp expression has been linked to MDR in a range of cancers for over 40 years, as well as a lack of chemotherapeutic responsiveness and a poor prognosis in breast and ovarian tumours. P-gp overexpression in cancer cells decreases chemotherapeutic accumulation and leads to resistance to a variety of currently available anti-cancer drugs, including vinca alkaloids (vinblastine), taxanes (paclitaxel), and anthracyclines (daunorubicin). The capacity of P-gp to transport such a broad range of chemical classes is ascribed, at least in part, to the multiple transport channels discovered inside the protein using molecular dynamics simulations [111]. Depending on the tissue of origin, studies show that P-gp overexpression in malignancies might be innate or acquired during pharmacological therapy. MDR inhibitor clinical studies have had mixed results, although the approach’s promise was revealed in research that employed cyclosporine to block P-gp in persons with low-risk AML [114]. Incorporating the inhibitor into therapy considerably increased relapse-free and overall survival. As previously indicated, the most prevalent clinical trial challenges were inhibitor toxicity, medication interactions, and questions about clinical trial design. None of these factors, however, lessen the significance or impact of effective P-gp inhibitors in cancer chemotherapies on patient outcomes [115].

P-gp and its drug resistance are major game-changers in the field of cancer. The drug metabolism, intake, pharmacodynamics, and pharmacokinetic characteristics mainly depend on these parameters. Researchers are working on a scenario to provide a better outbreak in the field of cancer by altering the issues associated with P-gp. Caution is recommended when manipulating P-gp doses because its inactivation will lead to negative impacts on our bodies [116].

For better treatment options, better studies on P-gp and its effects are needed. Orally administered drugs that are substrates to P-gp are not absorbed or show their physiological action as the stomach and small intestine show higher expressions of P-gp [117]. The plasma membrane of intestinal epithelial cells pumps back the drugs that enter the cells, which are recognized as substrates and excreted. We have shown that they play an important role in multidrug resistance in cancer, and this is also true in normal cellular pathogens, including protozoans and bacteria. This kind of transport system proves to be rather important, and in this review, we have raised and addressed the issues of the role of P-gp and drug transporters in cancer. Current indicators are that 50% of human cancers express P-gp at levels sufficient to confer multidrug resistance. There is an optimistic view that most of the drug resistance can be reversed by P-gp inhibitors. However, progress so far is slow. Most P-gp inhibitors are tested and show transient or no responses, wasting both money and time. Therefore, it is necessary to study P-gp and drug transporters to learn more about how the drugs can be handled in the body.

## 9. Conclusions

Cancer therapy remains difficult despite the use of multidrug regimens because tumour cells develop resistance quickly. Over the last three decades, the role of P-gp in chemo-resistance has been well established. However, to develop P-gp-specific inhibitors, researchers must first gain a better understanding of tissue distribution, cell-type specificity, immune side effects, body distribution/toxicity, and cell-specific cytotoxicity. It appears that avoiding P-gp-mediated efflux by synthetically modifying existing chemotherapeutic drugs is a very difficult drug discovery task. Understanding the 3D crystal structure of the P-gp protein provided new insights into drug-design strategies. Combining antitumor immune cells with P-gp inhibitors may improve tumour cell drug sensitivity while decreasing effective antitumor immune cell infiltration, complicating matters further. To effectively use P-gp inhibitors, these changes in the tumour microenvironment will necessitate more in-depth research in the future.

## Figures and Tables

**Table 1:** Substrates and inhibitors.

P-gp Substrates	P-gp Inhibitors
Vinblastine, mitorrycin c, etoposide, paclitaxel, topotecan, vincristine, actinomycin d, daunorubicin, doxorubicin, epirubicin, mitoxantrone	Cyclosporin a, verapamil, tamoxifen, quinine, megestrol acetate, fluperifixol, azopinin, valspoder, tesmiferene, dofequidar, tariquidar

**Table 2:** Clinical study reports of the action of P-gp on cancer cells.

Responsible Party	Phase	Condition or Disease	Treatment or Intervention	Number of Participants	Age and Sex	Outcome Measures	Study Start Date	Identification Number
Astellas Pharma Inc.	Phase 1	Prostate cancer	Enzalutamide, enzalutamide placebo, digoxin, rosuvastatin	24	18 years (Y) and older males	Pharmacokinetics and adverse effects	27 January 2020	NCT04094519
National Institute of Diabetes and Digestive and Kidney Diseases	Phase 2	Thyroid cancer	Procedure: skin biopsy	20	All	Thyroid hormone effect on P-gp activity in thyroid patients	April 2004	NCT00080574
National Institutes of Health (NIH) Clinical Center (CC)	Phase 2	Adrenal cortex neoplasm	Drug: XR9576 (tariquidar)	50	18 Y and older, all gender	Effect of P-gp antagonist before and after surgery.	October 2003	NCT00071058
Janssen-Cilag International NV	Phase 1	Healthy	Pimodivir, cyclosporine	18 participants	18 Y to 55 Y, all gender	Examine the pharmacokinetics of pimodivir in healthy adults using cyclosporine, a P-gp, BCRP, and organic-anion-transporting polypeptide Inhibitor.	6 December 2018	NCT03768609
National Cancer Institute (NCI)	Phase 2	Lung cancer, ovarian cancer cervix cancer, renal cancer	Docetaxel, tariquidar, 99mTc-sestamibi imaging	48 participants	18 Y and older, all gender	Tariquidar (XR9576), a P-gp antagonist, in combination with docetaxel in patients with ovarian, lung, renal, or cervical cancer: a study of the tariquidar-docetaxel interaction	September 2003	NCT00069160
Janssen Research & Development, LLC	Phase 1	Alzheimer disease	Drug: 11C-JNJ-63779586	11 participants	18 Y to 85 Y, all gender	P-gp and BCRP substrates, as well as the positron emission tomography ligand 11C-JNJ-63779586, were used in an open-label microdosing study in the human brain to investigate the regional brain kinetics of brain drug transporters.	17 May 2017	NCT03089918
NIH CC	Phase 1	BC, lung cancer, ovarian cancer	Vinorelbine, XR9576	30 participants	All age groups and gender	A clinical trial of the P-gp antagonist, XR9576, in combination with vinorelbine in patients with cancer: analysis of the interaction between XR9576 and vinorelbine	December 1999	NCT00001944
NIH CC	Phase 1	BC, kidney cancer, lymphoma cancer, metastasis ovarian cancer.	Drug: PSC 833	80 participants	All age groups and gender	A phase I study of P-gp antagonist PSC 833 infusional chemotherapy	September 1992	NCT00001302
NIH CC	Phase 1	Wilms’ tumour, sarcoma adenaocortical carcinoma, refractory cancer, coldrhood cancer	Tariquidar	29 participants	2 Y to 18 Y, all gender.	Tariquidar (XR9576), a P-gp inhibitor, was studied in a phase I trial and pharmacokinetic study in paediatric patients with refractory solid tumours, including brain tumours, in combination with doxorubicin, vinorelbine, or docetaxel.	February 15, 2001	NCT00011414
NIH CC	Phase 1	BC, carcinoma, renal cell lymphoma, ovarian cancer	PSC 833, paclitaxel	52 participants	All ages and all gender groups included	Paclitaxel infusion with the P-gp antagonist PSC 833 in a phase I study	March 1994	NCT00001383
Susan Bates, National Cancer Institute (NCI)	Not applicable	Cervical, ovarian, lung, breast, and renal cancer	Paclitaxel, CBT-1(Registered trademark), radiation: Tc 99m sestamibi	12 participants	18 Y to 80 Y, all sex eligible for the study	A pharmacodynamic study of CBT-1, a P-gp (Pgp) antagonist, evaluating Pgp inhibition in tumours and normal tissues	December 2007	NCT00972205
NIH CC	Phase 1	Kidney cancer and metastasis	PSC 833, vinblastine	46 participants	All ages and sex are eligible for the study.	Continuous intravenous PSC 833 and vinblastine infusion in patients with metastatic renal cancer: A phase I study	February 1997	NCT00001570
Francisco Robert, MD, the University of Alabama at Birmingham	Phase 2	Non-small cell lung cancer (NSCLC) stage IV NSCLC metastatic NSCLC	Cabazitaxel-XRP6258 (3-week cycle), cabazitaxel-XRP6258 (5-week cycle)	28 participants	19 Y and older and all sexes eligible for the study	A Phase II trial of a novel taxane (cabazitaxel-XRP6258) in patients with previously treated advanced non-small-cell lung cancer (NSCLC).	September 2011	NCT01438307
Merck KGaA, Darmstadt, Germany	Phase 1	Healthy	Dabigatran etexilate, tepotinib	20 participants	18 Y to 44 Y, all sexes are eligible for this study	The purpose of this Phase I, open-label, single-sequence, two-period study is to assess the effect of tepotinib on P-gp by investigating the pharmacokinetics of the P-gp probe substrate dabigatran etexilate in healthy subjects.	17 May 2018	NCT03492437
NIH CC		Low-grade glioma, glioblastoma multiforme, anaplastic astrocytoma, anaplastic oligodendroglioma, oligoastrocytoma		2 participants	18 Y to 99 Y, all sexes eligible for the study	(11C)N-desmethyl-loperamide as a P-gp function marker in glioma patients	13 January 2011	NCT01281982
Christopher H. Lowrey, Dartmouth-Hitchcock Medical Center	Phase 1	Leukaemia	Biological: sargramostim, Drug: mitomycin C, Drug: mitoxantrone hydrochloride	29 participants	18 Y to 120 Y, all sexes are eligible for this study.	A phase I evaluation of mitomycin C and mitoxantrone in patients with acute myelogenous leukaemia and a pilot clinical trial of mitomycin C modulation of MDR protein	September 1996	NCT00003003
Grupo Espanol de Investigacion en Sarcomas		Osteosarcoma		115 participants	12 Y to 30 Y, all sexes eligible for the study	P-gp expression as a biomarker for non-metastatic osteosarcoma of the extremities: a prospective observational study	2 July 2014	NCT04383288
Italian Sarcoma Group	Phase 2	Osteosarcoma	Mifamurtide arm, other: 3 drugs arm	225 participants	Up to 40 Y, all sexes are eligible	ABCB1/P-gp expression as a biologic stratification factor in patients with nonmetastatic osteosarcoma (ISG/OS-2)	23 June 2011	NCT01459484

## Data Availability

The data supporting the findings of this study are available within the article.

## References

[1] Husain A., Makadia V., Valicherla G.R., Riyazuddin M., Gayen J.R. (2022). Approaches to Minimize the Effects of P-glycoprotein in Drug Transport: A Review. Drug Dev. Res..

[2] Wright S.C.E., Vasilevski N., Serra V., Rodon J., Eichhorn P.J.A. (2021). Mechanisms of Resistance to PI3K Inhibitors in Cancer: Adaptive Responses, Drug Tolerance and Cellular Plasticity. Cancers.

[3] Aissa A.F., Islam A.B., Ariss M.M., Go C.C., Rader A.E., Conrardy R.D., Gajda A.M., Rubio-Perez C., Valyi-Nagy K., Pasquinelli M. (2021). Single-Cell Transcriptional Changes Associated with Drug Tolerance and Response to Combination Therapies in Cancer. Nat. Commun..

[4] Strand V., Goncalves J., Isaacs J.D. (2021). Immunogenicity of Biologic Agents in Rheumatology. Nat. Rev. Rheumatol..

[5] Zuo H.-L., Huang H.-Y., Lin Y.-C.-D., Cai X.-X., Kong X.-J., Luo D.-L., Zhou Y.-H., Huang H.-D. (2022). Enzyme Activity of Natural Products on Cytochrome P450. Molecules.

[6] Bluth M.H., Pincus M.R., Abraham N.Z. (2021). Toxicology and Therapeutic Drug Monitoring. Henry’s Clinical Diagnosis and Management by Laboratory Methods E-Book.

[7] Estevinho M.M., Fernandes C., Silva J.C., Gomes A.C., Afecto E., Correia J., Carvalho J. (2022). Role of ATP-Binding Cassette Transporters in Sorafenib Therapy for Hepatocellular Carcinoma: An Overview. Curr. Drug Targets.

[8] Cavalu S., Antoniac I.V., Mohan A., Bodog F., Doicin C., Mates I., Ulmeanu M., Murzac R., Semenescu A. (2020). Nanoparticles and Nanostructured Surface Fabrication for Innovative Cranial and Maxillofacial Surgery. Materials.

[9] Islam M.R., Islam F., Nafady M.H., Akter M., Mitra S., Das R., Urmee H., Shohag S., Akter A., Chidambaram K. (2022). Natural Small Molecules in Breast Cancer Treatment: Understandings from a Therapeutic Viewpoint. Molecules.

[10] Kumar S., Kushwaha P.P., Gupta S. (2019). Emerging Targets in Cancer Drug Resistance. Cancer Drug Resist..

[11] Srikant S. (2020). Evolutionary History of ATP-binding Cassette Proteins. FEBS Lett..

[12] Karthika C., Hari B., Rahman M.H., Akter R., Najda A., Albadrani G.M., Sayed A.A., Akhtar M.F., Abdel-Daim M.M. (2021). Multiple Strategies with the Synergistic Approach for Addressing Colorectal Cancer. Biomed. Pharmacother..

[13] Behl T., Kaur I., Sehgal A., Kumar A., Uddin M., Bungau S. (2021). The Interplay of ABC Transporters in Aβ Translocation and Cholesterol Metabolism: Implicating Their Roles in Alzheimer’s Disease. Mol. Neurobiol..

[14] Karthika C., Sureshkumar R. (2020). P-Glycoprotein Efflux Transporters and Its Resistance Its Inhibitors and Therapeutic Aspects.

[15] Karthika C., Sureshkumar R. (2019). Can Curcumin along with Chemotherapeutic Drug and Lipid Provide an Effective Treatment of Metastatic Colon Cancer and Alter Multidrug Resistance?. Med. Hypotheses.

[16] Karthika C., Sureshkumar R. (2019). Formulation and in-vitro characterization of 5-fluorouracil and flavonoid dual lipid drug conjugates loaded self nanomulsifying drug delivery system for cancer targeting. Int. J. Pharm. Sci. Res..

[17] Pyun J., McInnes L.E., Donnelly P.S., Mawal C., Bush A.I., Short J.L., Nicolazzo J.A. (2022). Copper Bis (Thiosemicarbazone) Complexes Modulate P-glycoprotein Expression and Function in Human Brain Microvascular Endothelial Cells. J. Neurochem..

[18] Robinson K., Tiriveedhi V. (2020). Perplexing Role of P-Glycoprotein in Tumor Microenvironment. Front. Oncol..

[19] Patil K., Khan F.B., Akhtar S., Ahmad A., Uddin S. (2021). The Plasticity of Pancreatic Cancer Stem Cells: Implications in Therapeutic Resistance. Cancer Metastasis Rev..

[20] Juan-Carlos P.-D.M., Perla-Lidia P.-P., Stephanie-Talia M.-M., Mónica-Griselda A.-M., Luz-María T.-E. (2021). ABC Transporter Superfamily. An Updated Overview, Relevance in Cancer Multidrug Resistance and Perspectives with Personalized Medicine. Mol. Biol. Rep..

[21] Alemayehu D., Melisie G., Taye K., Tadesse E. (2019). The Role of ABC Efflux Transporter in Treatment of Pharmaco-Resistant Schizophrenia: A Review Article. Clin. Pharmacol. Biopharm..

[22] Cecchin E., De Mattia E., Ecca F., Toffoli G. (2018). Host Genetic Profiling to Increase Drug Safety in Colorectal Cancer from Discovery to Implementation. Drug Resist. Updates.

[23] Hsin C., Stoffel M.S., Gazzaz M., Schaeffeler E., Schwab M., Fuhr U., Taubert M. (2020). Combinations of Common SNPs of the Transporter Gene ABCB1 Influence Apparent Bioavailability, but Not Renal Elimination of Oral Digoxin. Sci. Rep..

[24] Marinko J.T., Huang H., Penn W.D., Capra J.A., Schlebach J.P., Sanders C.R. (2019). Folding and Misfolding of Human Membrane Proteins in Health and Disease: From Single Molecules to Cellular Proteostasis. Chem. Rev..

[25] Nurmatova S., Kapralova Y., Abdurakhimov A., Turdikulova S., Dalimova D. (2022). Determination of the Frequency of ABCB1 Gene Polymorphisms (C1236T, C3435T) in the Population of the Tashkent Region of Uzbekistan. Biochem. Biotechnol. Res..

[26] Sindhu R.K., Najda A., Kaur P., Shah M., Singh H., Kaur P., Cavalu S., Jaroszuk-Sierocińska M., Rahman M. (2021). Potentiality of Nanoenzymes for Cancer Treatment and Other Diseases: Current Status and Future Challenges. Materials.

[27] Liu X. (2019). ABC Family Transporters. Drug Transporters in Drug Disposition, Effects and Toxicity.

[28] Chung F.S., Santiago J.S., De Jesus M.F.M., Trinidad C.V., See M.F.E. (2016). Disrupting P-Glycoprotein Function in Clinical Settings: What Can We Learn from the Fundamental Aspects of This Transporter?. Am. J. Cancer Res..

[29] Fu D. (2013). Where Is It and How Does It Get There–Intracellular Localization and Traffic of P-Glycoprotein. Front. Oncol..

[30] Guy-Viterbo V. (2019). Toward Individualization Regimen of Tacrolimus in Pediatric Liver Transplantation. Ph.D. Thesis.

[31] Biedler J.L., Spengler B.A. (1994). Reverse Transformation of Multidrug-Resistant Cells. Cancer Metastasis Rev..

[32] Wang P., Hou Y., Zhang W., Zhang H., Che X., Gao Y., Liu Y., Yang D., Wang J., Xiang R. (2019). Pseudoginsenoside-F11 Attenuates Lipopolysaccharide-Induced Acute Lung Injury by Suppressing Neutrophil Infiltration and Accelerating Neutrophil Clearance. Inflammation.

[33] Czuba L.C., Hillgren K.M., Swaan P.W. (2018). Post-Translational Modifications of Transporters. Pharmacol. Ther..

[34] Kapoor K., Pant S., Tajkhorshid E. (2021). Active Participation of Membrane Lipids in Inhibition of Multidrug Transporter P-Glycoprotein. Chem. Sci..

[35] Xia D., Zhou F., Esser L. (2019). Emerging Consensus on the Mechanism of Polyspecific Substrate Recognition by the Multidrug Transporter P-Glycoprotein. Cancer Drug Resist..

[36] Clouser A.F., Atkins W.M. (2022). Long Range Communication between the Drug-Binding Sites and Nucleotide Binding Domains of the Efflux Transporter ABCB1. Biochemistry.

[37] Chakravarty M., Vora A. (2021). Nanotechnology-Based Antiviral Therapeutics. Drug Deliv. Transl. Res..

[38] Foronda M. (2021). Investigating the Mechanism of the Escherichia Coli ATP-Binding Cassette (ABC) Transporter MetNI. Master’s Thesis.

[39] Kodan A., Futamata R., Kimura Y., Kioka N., Nakatsu T., Kato H., Ueda K. (2021). ABCB1/MDR1/P-gp Employs an ATP-dependent Twist-and-squeeze Mechanism to Export Hydrophobic Drugs. FEBS Lett..

[40] Taylor J., Bebawy M. (2019). Proteins Regulating Microvesicle Biogenesis and Multidrug Resistance in Cancer. Proteomics.

[41] Alam A., Küng R., Kowal J., McLeod R.A., Tremp N., Broude E.V., Roninson I.B., Stahlberg H., Locher K.P. (2018). Structure of a Zosuquidar and UIC2-Bound Human-Mouse Chimeric ABCB1. Proc. Natl. Acad. Sci. USA.

[42] Dunk C.E., Pappas J.J., Lye P., Kibschull M., Javam M., Bloise E., Lye S.J., Szyf M., Matthews S.G. (2018). P-Glycoprotein (P-gp)/ABCB 1 Plays a Functional Role in Extravillous Trophoblast (EVT) Invasion and Is Decreased in the Pre-eclamptic Placenta. J. Cell. Mol. Med..

[43] Gaohua L., Miao X., Dou L. (2021). Crosstalk of Physiological PH and Chemical PKa under the Umbrella of Physiologically Based Pharmacokinetic Modeling of Drug Absorption, Distribution, Metabolism, Excretion, and Toxicity. Expert Opin. Drug Metab. Toxicol..

[44] Vaidya F.U., Sufiyan Chhipa A., Mishra V., Gupta V.K., Rawat S.G., Kumar A., Pathak C. (2020). Molecular and Cellular Paradigms of Multidrug Resistance in Cancer. Cancer Rep..

[45] Testa U., Castelli G., Pelosi E. (2019). Cellular and Molecular Mechanisms Underlying Prostate Cancer Development: Therapeutic Implications. Medicines.

[46] Khatoon E., Banik K., Harsha C., Sailo B.L., Thakur K.K., Khwairakpam A.D., Vikkurthi R., Devi T.B., Gupta S.C., Kunnumakkara A.B. (2020). Phytochemicals in Cancer Cell Chemosensitization: Current Knowledge and Future Perspectives. Seminars in Cancer Biology.

[47] Turner A.P., Alam C., Bendayan R. (2020). Efflux Transporters in Cancer Resistance: Molecular and Functional Characterization of P-Glycoprotein. Drug Efflux Pumps in Cancer Resistance Pathways: From Molecular Recognition and Characterization to Possible Inhibition Strategies in Chemotherapy.

[48] Guerra A.R., Duarte M.F., Duarte I.F. (2018). Targeting Tumor Metabolism with Plant-Derived Natural Products: Emerging Trends in Cancer Therapy. J. Agric. Food Chem..

[49] Erin N., Grahovac J., Brozovic A., Efferth T. (2020). Tumor Microenvironment and Epithelial Mesenchymal Transition as Targets to Overcome Tumor Multidrug Resistance. Drug Resist. Updates.

[50] Cavalu S., Simon V. (2007). Proteins adsorption to orthopaedic biomaterials: Vibrational spectroscopy evidence. J. Optoelectron. Adv. Mater..

[51] Gonçalves A.C., Richiardone E., Jorge J., Polónia B., Xavier C.P.R., Salaroglio I.C., Riganti C., Vasconcelos M.H., Corbet C., Sarmento-Ribeiro A.B. (2021). Impact of Cancer Metabolism on Therapy Resistance-Clinical Implications. Drug Resist. Updates.

[52] Nguyen T.-T.-L., Duong V.-A., Maeng H.-J. (2021). Pharmaceutical Formulations with P-Glycoprotein Inhibitory Effect as Promising Approaches for Enhancing Oral Drug Absorption and Bioavailability. Pharmaceutics.

[53] Mirzaei S., Gholami M.H., Hashemi F., Zabolian A., Farahani M.V., Hushmandi K., Zarrabi A., Goldman A., Ashrafizadeh M., Orive G. (2021). Advances in Understanding the Role of P-Gp in Doxorubicin Resistance: Molecular Pathways, Therapeutic Strategies, and Prospects. Drug Discov. Today.

[54] Bhattacharya T., Soares G.A.B.e., Chopra H., Rahman M.M., Hasan Z., Swain S.S., Cavalu S. (2022). Applications of Phyto-Nanotechnology for the Treatment of Neurodegenerative Disorders. Materials.

[55] Ge C., Xu D., Yu P., Fang M., Guo J., Qiao Y., Chen S., Wang H., Zhang Y. (2021). P-Gp Expression Inhibition Mediates Placental Glucocorticoid Barrier Opening and Fetal Weight Loss. BMC Med..

[56] Hartman J.H., Widmayer S.J., Bergemann C.M., King D.E., Morton K.S., Romersi R.F., Jameson L.E., Leung M.C.K., Andersen E.C., Taubert S. (2021). Xenobiotic Metabolism and Transport in Caenorhabditis Elegans. J. Toxicol. Environ. Health Part B.

[57] Zhou S., Chan E., Lim L.Y., Boelsterli U.A., Li S.C., Wang J., Zhang Q., Huang M., Xu A. (2004). Therapeutic Drugs That Behave as Mechanism-Based Inhibitors of Cytochrome P450 3A4. Curr. Drug Metab..

[58] Kunihara M., Nagai J., Murakami T., Takano M. (1998). Renal Excretion of Rhodamine 123, a P-Glycoprotein Substrate, in Rats with Glycerol-Induced Acute Renal Failure. J. Pharm. Pharmacol..

[59] Yang X. (2021). Effect of Combination of the Mammalian Lignan, Enterolactone, with Tyrosine kinase Inhibitors on Markers of Hepatic Fibrosis. Ph.D. Thesis.

[60] Leung L., Oganesian A. (2009). Introduction to Drug Transporters. Drug Metabolism Handbook: Concepts and Applications.

[61] El-Readi M.Z., Al-Abd A.M., Althubiti M.A., Almaimani R.A., Al-Amoodi H.S., Ashour M.L., Wink M., Eid S.Y. (2021). Multiple Molecular Mechanisms to Overcome Multidrug Resistance in Cancer by Natural Secondary Metabolites. Front. Pharmacol..

[62] Kang J.Y., Llewellyn E., Chen J., Olinares P.D.B., Brewer J., Chait B.T., Campbell E.A., Darst S.A. (2021). Structural Basis for Transcription Complex Disruption by the Mfd Translocase. eLife.

[63] Kanlikilicer P., Bayraktar R., Denizli M., Rashed M.H., Ivan C., Aslan B., Mitra R., Karagoz K., Bayraktar E., Zhang X. (2018). Exosomal MiRNA Confers Chemo Resistance via Targeting Cav1/p-Gp/M2-Type Macrophage Axis in Ovarian Cancer. EBioMedicine.

[64] Sajid A., Lusvarghi S., Ambudkar S.V. (2022). The P-glycoprotein multidrug transporter. Drug Transp. Mol. Charact. Role Drug Dispos..

[65] Koehn L.M. (2021). ABC Transporters: An Overview. ADME Encycl. A Compr. Guid. Biopharmacy Pharmacokinet..

[66] Yang F., Li A., Liu H., Zhang H. (2018). Gastric Cancer Combination Therapy: Synthesis of a Hyaluronic Acid and Cisplatin Containing Lipid Prodrug Coloaded with Sorafenib in a Nanoparticulate System to Exhibit Enhanced Anticancer Efficacy and Reduced Toxicity. Drug Des. Dev. Ther..

[67] Rowbottom C., Pietrasiewicz A., Tuczewycz T., Grater R., Qiu D., Kapadnis S., Trapa P. (2021). Optimization of Dose and Route of Administration of the P-glycoprotein Inhibitor, Valspodar (PSC-833) and the P-glycoprotein and Breast Cancer Resistance Protein Dual-inhibitor, Elacridar (GF120918) as Dual Infusion in Rats. Pharmacol. Res. Perspect..

[68] Rehman S.K., Haynes J., Collignon E., Brown K.R., Wang Y., Nixon A.M.L., Bruce J.P., Wintersinger J.A., Mer A.S., Lo E.B.L. (2021). Colorectal Cancer Cells Enter a Diapause-like DTP State to Survive Chemotherapy. Cell.

[69] Ara N., Sultana T., Bolleddu R., Venkatesh S., Kiran A.R. (2021). A Strategy to Enhance Bioavailability of Drug Candidates: Natural Bioenhancers. Int. J. Pharm. Bio Med. Sci..

[70] Verma R., Kaushik A., Almeer R., Habibur Rahman M., Abdel-Daim M.M., Kaushik D. (2021). Improved Pharmacodynamic Potential of Rosuvastatin by Self-Nanoemulsifying Drug Delivery System: An in Vitro and in Vivo Evaluation. Int. J. Nanomedicine.

[71] Wong I.L.K., Wang X., Liu Z., Sun W., Li F., Wang B., Li P., Wan S., Chow L.M.C. (2021). Synthesis and Evaluation of Stereoisomers of Methylated Catechin and Epigallocatechin Derivatives on Modulating P-Glycoprotein-Mediated Multidrug Resistance in Cancers. Eur. J. Med. Chem..

[72] Rosser S.P.A., Atkinson C., Nath C.E., Fletcher J.I. (2022). Quantification of Vincristine and Tariquidar by LC-MS/MS in Mouse Whole Blood Using Volumetric Absorptive Microsampling for Pharmacokinetic Applications. J. Sep. Sci..

[73] Poma P., Labbozzetta M., Notarbartolo M. (2021). Patterns of Innate or Acquired Resistance to Anticancer Drugs: Our Experience to Overcome It. Crit. Rev. Oncog..

[74] Chevyreva V. (2021). Study of Transporter and Tight Junction Modification during Ageing, Cerebral Amyloid Angiopathy and Ischaemia at the Blood-Brain Barrier. Ph.D. Thesis.

[75] Fan Y.-F., Zhang W., Zeng L., Lei Z.-N., Cai C.-Y., Gupta P., Yang D.-H., Cui Q., Qin Z.-D., Chen Z.-S. (2018). Dacomitinib Antagonizes Multidrug Resistance (MDR) in Cancer Cells by Inhibiting the Efflux Activity of ABCB1 and ABCG2 Transporters. Cancer Lett..

[76] Karthika C., Sureshkumar R. (2021). Incorporation of Natural Assumption to Deal with Cancer. Environ. Sci. Pollut. Res. Int..

[77] Shan Y., Cen Y., Zhang Y., Tan R., Zhao J., Nie Z., Zhang J., Yu S. (2021). Effect of P-Glycoprotein Inhibition on the Penetration of Ceftriaxone Across the Blood–Brain Barrier. Neurochem. Res..

[78] Mora Lagares L., Pérez-Castillo Y., Minovski N., Novič M. (2021). Structure-Function Relationships in the Human P-Glycoprotein (ABCB1): Insights from Molecular Dynamics Simulations. Int. J. Mol. Sci..

[79] Arnst K.E., Banerjee S., Chen H., Deng S., Hwang D., Li W., Miller D.D. (2019). Current Advances of Tubulin Inhibitors as Dual Acting Small Molecules for Cancer Therapy. Med. Res. Rev..

[80] Kyeremateng J. (2021). Characterization of Cucurbitacin-Inspired Estrone Analogues as Novel Inhibitors of Human ATP-Binding Cassette Proteins (ABCB1 and ABCC1).

[81] Labbozzetta M., Poma P., Tutone M., McCubrey J.A., Sajeva M., Notarbartolo M. (2022). Phytol and Heptacosane Are Possible Tools to Overcome Multidrug Resistance in an In Vitro Model of Acute Myeloid Leukemia. Pharmaceuticals.

[82] Elefantova K., Lakatos B., Kubickova J., Sulova Z., Breier A. (2018). Detection of the Mitochondrial Membrane Potential by the Cationic Dye JC-1 in L1210 Cells with Massive Overexpression of the Plasma Membrane ABCB1 Drug Transporter. Int. J. Mol. Sci..

[83] Martins E., Silva V., Lemos A., Palmeira A., Puthongking P., Sousa E., Rocha-Pereira C., Ghanem C.I., Carmo H., Remião F. (2019). Newly Synthesized Oxygenated Xanthones as Potential P-Glycoprotein Activators: In Vitro, Ex Vivo, and in Silico Studies. Molecules.

[84] Brahmachari G. (2021). Epothilones A and B: The 16-Membered Natural Macrolides as a Fascinating Template for Antibreast Cancer Drug Discovery. Discovery and Development of Anti-Breast Cancer Agents from Natural Products.

[85] Asati V. (2022). Perspectives of Anti-Cancer Phytoconstituents in Pharmacotherapy. Int. J. Med. Pharm. Sci..

[86] Dhupal M., Chowdhury D. (2020). Phytochemical-Based Nanomedicine for Advanced Cancer Theranostics: Perspectives on Clinical Trials to Clinical Use. Int. J. Nanomed..

[87] Kano H., Kondziolka D., Zorro O., Lobato-Polo J., Flickinger J.C., Lunsford L.D. (2009). The Results of Resection after Stereotactic Radiosurgery for Brain Metastases: Clinical Article. J. Neurosurg..

[88] Rachmale M., Rajput N., Jadav T., Sahu A.K., Tekade R.K., Sengupta P. (2022). Implication of Metabolomics and Transporter Modulation Based Strategies to Minimize Multidrug Resistance and Enhance Site-Specific Bioavailability: A Needful Consideration toward Modern Anticancer Drug Discovery. Drug Metab. Rev..

[89] Martino E., Casamassima G., Castiglione S., Cellupica E., Pantalone S., Papagni F., Rui M., Siciliano A.M., Collina S. (2018). Vinca Alkaloids and Analogues as Anti-Cancer Agents: Looking Back, Peering Ahead. Bioorg. Med. Chem. Lett..

[90] Kou L., Sun R., Bhutia Y.D., Yao Q., Chen R. (2018). Emerging Advances in P-Glycoprotein Inhibitory Nanomaterials for Drug Delivery. Expert Opin. Drug Deliv..

[91] Alavi M., Hamidi M. (2019). Passive and Active Targeting in Cancer Therapy by Liposomes and Lipid Nanoparticles. Drug Metab. Pers. Ther..

[92] Xenaki K.T., Dorrestijn B., Muns J.A., Adamzek K., Doulkeridou S., Houthoff H., Oliveira S., van Bergen Henegouwen P.M.P. (2021). Homogeneous Tumor Targeting with a Single Dose of HER2-Targeted Albumin-Binding Domain-Fused Nanobody-Drug Conjugates Results in Long-Lasting Tumor Remission in Mice. Theranostics.

[93] Yang L., Xie X., Tu Z., Fu J., Xu D., Zhou Y. (2021). The Signal Pathways and Treatment of Cytokine Storm in COVID-19. Signal Transduct. Target. Ther..

[94] Jabbour E., Paul S., Kantarjian H. (2021). The Clinical Development of Antibody–Drug Conjugates—Lessons from Leukaemia. Nat. Rev. Clin. Oncol..

[95] Mobasheri T., Rayzan E., Shabani M., Hosseini M., Mahmoodi Chalbatani G., Rezaei N. (2021). Neuroblastoma-targeted Nanoparticles and Novel Nanotechnology-based Treatment Methods. J. Cell. Physiol..

[96] Huang X., Hussain B., Chang J. (2021). Peripheral Inflammation and Blood–Brain Barrier Disruption: Effects and Mechanisms. CNS Neurosci. Ther..

[97] Wang Y.-H., Imai Y., Shiseki M., Tanaka J., Motoji T. (2018). Knockdown of the Wnt Receptor Frizzled-1 (FZD1) Reduces MDR1/P-Glycoprotein Expression in Multidrug Resistant Leukemic Cells and Inhibits Leukemic Cell Proliferation. Leuk. Res..

[98] Miere F., Fritea L., Cavalu S., Vicas S.I. (2020). Formulation, characterization, and advantages of using liposomes in multiple therapies. Pharmacophore.

[99] Luo H., Cao G., Luo C., Tan D., Vong C.T., Xu Y., Wang S., Lu H., Wang Y., Jing W. (2022). Emerging Pharmacotherapy for Inflammatory Bowel Diseases. Pharmacol. Res..

[100] Mishra J., Stubbs M., Kuang L., Vara N., Kumar P., Kumar N. (2022). Inflammatory Bowel Disease Therapeutics: A Focus on Probiotic Engineering. Mediat. Inflamm..

[101] Deng R., Fan F., Yi H., Liu F., He G., Sun H., Su Y. (2019). MEG3 Affects the Progression and Chemoresistance of T-cell Lymphoblastic Lymphoma by Suppressing Epithelial-mesenchymal Transition via the PI3K/MTOR Pathway. J. Cell. Biochem..

[102] Zhu M., Wang S. (2021). Functional Nucleic-Acid-Decorated Spherical Nanoparticles: Preparation Strategies and Current Applications in Cancer Therapy. Small Sci..

[103] Mao X., Xu J., Wang W., Liang C., Hua J., Liu J., Zhang B., Meng Q., Yu X., Shi S. (2021). Crosstalk between Cancer-Associated Fibroblasts and Immune Cells in the Tumor Microenvironment: New Findings and Future Perspectives. Mol. Cancer.

[104] Abd El-Fattah E.E., Saber S., Mourad A.A.E., El-Ahwany E., Amin N.A., Cavalu S., Yahya G., Saad A.S., Alsharidah M., Shata A. (2022). The Dynamic Interplay between AMPK/NFκB Signaling and NLRP3 Is a New Therapeutic Target in Inflammation: Emerging Role of Dapagliflozin in Overcoming Lipopolysaccharide-Mediated Lung Injury. Biomed. Pharmacother..

[105] Jorgovanovic D., Song M., Wang L., Zhang Y. (2020). Roles of IFN-γ in Tumor Progression and Regression: A Review. Biomark. Res..

[106] Jahan S., Mukherjee S., Ali S., Bhardwaj U., Choudhary R.K., Balakrishnan S., Naseem A., Mir S.A., Banawas S., Alaidarous M. (2022). Pioneer Role of Extracellular Vesicles as Modulators of Cancer Initiation in Progression, Drug Therapy, and Vaccine Prospects. Cells.

[107] De Vera A.A., Gupta P., Lei Z., Liao D., Narayanan S., Teng Q., Reznik S.E., Chen Z.-S. (2019). Immuno-Oncology Agent IPI-549 Is a Modulator of P-Glycoprotein (P-Gp, MDR1, ABCB1)-Mediated Multidrug Resistance (MDR) in Cancer: In Vitro and in Vivo. Cancer Lett..

[108] Cui Q., Cai C.-Y., Gao H.-L., Ren L., Ji N., Gupta P., Yang Y., Shukla S., Ambudkar S.V., Yang D.-H. (2019). Glesatinib, a c-MET/SMO Dual Inhibitor, Antagonizes P-Glycoprotein Mediated Multidrug Resistance in Cancer Cells. Front. Oncol..

[109] Fatima M., Iqubal M.K., Iqubal A., Kaur H., Gilani S.J., Rahman M.H., Ahmadi A., Rizwanullah M. (2021). Current Insight into the Therapeutic Potential of Phytocompounds and Their Nanoparticle-Based Systems for Effective Management of Lung Cancer. Anticancer Agents Med. Chem..

[110] Karthika C., Sureshkumar R., Zehravi M., Akter R., Ali F., Ramproshad S., Mondal B., Kundu M.K., Dey A., Rahman H. (2022). Multidrug Resistance in Cancer Cells: Focus on a Possible Strategy Plan to Address Colon Carcinoma Cells. Life.

[111] Jin H., Zhu Y., Wang C., Meng Q., Wu J., Sun P., Ma X., Sun H., Huo X., Liu K. (2020). Molecular Pharmacokinetic Mechanism of the Drug-Drug Interaction between Genistein and Repaglinide Mediated by P-Gp. Biomed. Pharmacother..

[112] Laiolo J., Lanza P.A., Parravicini O., Barbieri C., Insuasty D., Cobo J., Vera D., Enriz R.D., Carpinella M.C. (2021). Structure Activity Relationships and the Binding Mode of Quinolinone-Pyrimidine Hybrids as Reversal Agents of Multidrug Resistance Mediated by P-Gp. Sci. Rep..

[113] Wang S., Teng Q., Wang S., Lei Z., Hu H., Lv H., Chen B., Wang J., Shi X., Xu W. (2022). Preclinical Studies of the Triazolo [1, 5-a] Pyrimidine Derivative WS-716 as a Highly Potent, Specific and Orally Active P-Glycoprotein (P-Gp) Inhibitor. Acta Pharm. Sin. B.

[114] Zhang H., Xu H., Ashby Jr C.R., Assaraf Y.G., Chen Z., Liu H. (2021). Chemical Molecular-based Approach to Overcome Multidrug Resistance in Cancer by Targeting P-glycoprotein (P-gp). Med. Res. Rev..

[115] Li M., Yin D., Li J., Shao F., Zhang Q., Jiang Q., Zhang M., Yang Y. (2020). Rosmarinic Acid, the Active Component of Salvia Miltiorrhizae, Improves Gliquidone Transport by Regulating the Expression and Function of P-Gp and BCRP in Caco-2 Cells. Pharm. Int. J. Pharm. Sci..

[116] Stage T.B., Mortensen C., Khalaf S., Steffensen V., Hammer H.S., Xiong C., Nielsen F., Poetz O., Svenningsen Å.F., Rodriguez-Antona C. (2020). P-Glycoprotein Inhibition Exacerbates Paclitaxel Neurotoxicity in Neurons and Patients with Cancer. Clin. Pharmacol. Ther..

[117] Nguyen H.-M., Guz-Montgomery K., Lowe D.B., Saha D. (2021). Pathogenetic Features and Current Management of Glioblastoma. Cancers.

